# Metronome-guided training accelerates the adaptation to an aerobic training pace in swimming

**DOI:** 10.3389/fspor.2025.1588758

**Published:** 2025-05-09

**Authors:** Marco Fassone, Luca Puce, Monica Biggio, Laura Avanzino, Marco Bove, Ambra Bisio

**Affiliations:** ^1^ Department of Neuroscience, Rehabilitation, Ophthalmology, Genetics and Maternal Child Health, Università Degli Studi di Genova, Genoa, Italy; ^2^ Department of Experimental Medicine, Section of Human Physiology, Università Degli Studi di Genova, Genoa, Italy; ^3^ IRCCS Ospedale Policlinico San Martino, Genoa, Italy; ^4^ Centro Polifunzionale di Scienze Motorie, Università Degli Studi di Genova, Genoa, Italy

**Keywords:** metronome, pacing, swimming, learning, motor timing, training

## Abstract

Swimmers often use metronomes during training, but their effectiveness in learning a new swimming pace is unclear. Adapting motor skills to new timing is complex and occurs in stages. This study investigated these stages by assessing the ability of swimmers to reproduce a learned pace without a metronome. In total, 24 participants were divided into two groups. The main group completed 4 days of training/testing and 3 follow-up days. Each training day included a 100 m front crawl trial without a metronome, followed by three 2 × 100 m training sets with metronome synchronization and a further 100 m test without the metronome. The performance measures were total 100 m time, 25 m lap times, temporal errors over 100 m (Err100) and 25 m (Err25), and the coefficient of variation of Err25 (CV). The control group followed the same protocol, but only for 2 days. Results showed that in the main group, Err100, Err25, and CV improved after the first training day and remained consistent throughout the week. Notably, Err25 and CV improvements persisted up to 10 days post-training. The control group showed no improvement after 2 days. These results suggest that metronome-guided training effectively helps swimmers adapt their pace to new motor timing, with effects lasting up to 10 days.

## 1 Introduction

In individual sports defined as “closed-loop design,” such as running, swimming, cycling, rowing, skiing, and speed skating (1), the aim of the athletes is to finish a known distance in the shortest time possible (2). These athletes have to compete against opponents in order to finish the race in a better time than the competitors (2). Consequently, the actions and strategies of opponents can influence the dynamics of the race, making coaching and individual tactics key elements to achieve success (3). Poor energy management, such as starting too fast or too slow, can significantly impair overall performance. Research has shown that maintaining a controlled and balanced pace is essential to achieve maximum efficiency and secure the best possible time (4). The ability to regulate one's pace, avoiding excessive speed drops in the final stages or overly aggressive initial phases, is a key determinant of success (5).

Improving energy expenditure, e.g., pacing, is therefore crucial for competitive swimmers (6). A large part of swim training focuses on enhancing aerobic endurance, defined as the ability to sustain a high percentage of VO2max over an extended period (7). Although aerobic capacity is typically emphasized in longer races, such as 400, 800, and 1,500 m (8, 9) and is essential in open water events (5, 10, and 25 km) (10, 11), its contribution should not be underestimated in shorter distances such as the 50, 100, and 200 m (12). Thus, aerobic training plays a vital role even in sprint events, providing swimmers with the capacity to sustain peak performance throughout the race (13). In particular, the 100-m swimming event, often perceived as predominantly anaerobic, has been shown to rely significantly on both glycolytic and aerobic energy systems (14). Studies in the literature indicate that, contrary to traditional views, the 100-m race requires the rapid activation of both energy pathways, along with the ability to sustain high lactate concentrations in the muscles (13, 14). During aerobic endurance interval training, pacing, the duration of the training set, the duration of each repetition, the number of repetitions, and the rest interval need to be defined and monitored during the season to avoid overloading or underloading (15, 16).

With the aim of enhancing endurance, work rate, and efficiency in cyclical sports, recent scientific studies have proposed exercises based on auditory-motor synchronization, which consists of adapting the spontaneous movement tempo to a new motor timing imposed by acoustic stimuli (17). This acquired motor timing would allow the new performance pace to be defined. Since people are predisposed to auditory-motor synchronization from an early age (18, 19), the use of acoustic cues represents a potential means of improving performance in practical settings, including rehabilitation (20), physical activity (21, 22), and sports (23). For these reasons, sport-specific audio devices such as the metronome are increasingly being used by athletes and coaches as a tool to impose a new pace on an already known sports skill (24). In recent years, the use of a metronome has been introduced into swimming training to improve stroke rate and tailor race strategy for optimal performance (13, 25). The rationale is that the metronome assists swimmers during training in learning to better manage energy expenditure, with the ultimate goal of optimizing strategy during competition. For instance, some studies have used the metronome to regulate swimming speed by adjusting the time between the audio feedback from the metronome to match the time it takes to swim from one side of the pool to the other (26–28).

In such a context, in order to plan the training sessions accurately, it is essential to know how long it takes the athlete to adapt to the new pace imposed by the acoustic stimulus. This adaptation process depends on the ability to compensate for environmental changes (29) (that, when using a metronome, requires synchronization with the auditory feedback) and is characterized by different stages: a “fast stage,” which occurs after the first training session; a “slow stage and consolidation,” in which further gains are made over several sessions, leading to the automatization of the gesture incorporating the new temporal features; and finally, a “retention stage,” which occurs when the adapted motor skill can thus be easily performed despite long periods without practice (29–31).

Although understanding how long swimmers take to learn the optimal training pace is crucial for performance improvement, no study has assessed this aspect. Therefore, the main experiment in this study aimed to determine how many training sessions swimmers require to adapt to an individually defined pace set by a metronome. Based on the results of a previous motor learning study (32), one may expect that a single training session would suffice to induce this adaptation. However, this is not certain, as the movement involved is significantly more complex than that examined by Bonassi et al. Furthermore, a control experiment was included to compare the time required to adapt to the new pace using the metronome vs. traditional swimming training, in which the coach provides time feedback at the end of each repetition. This insight will help evaluate the practical effectiveness of using a metronome in swimming training.

## 2 Materials and methods

### 2.1 Participants

To determine the appropriate sample size, a power analysis was conducted using G*Power 3.1.9.7 (Düsseldorf, Germany) (33). The power analysis was based on an *F*-test (ANOVA: repeated measures, within factor), assuming a high effect size (*f* = 0.4) and a desired power of 0.80. This indicates that 12 participants were required to detect a significant effect.

In total, 24 competitive swimmers (13 men and 11 women) volunteered to participate in this study, with 12 participants assigned to the main experiment (6 men and 6 women) and 12 to the control experiment (7 men and 5 women). The two groups were homogeneous in terms of age, anthropometric characteristics, and level of experience in swimming (Table 1). According to McKay et al. (34), these athletes can be classified as being at the trained/developmental level. All of them specialized in freestyle and competed at the regional and national levels. All the swimmers followed the same training regimen, as they were part of the same team. Specifically, they were in a specific phase of the season, and before the experimental session, their training focused on preparing for the 100 and 200 m freestyle competitions.

**Table 1:** Characteristics of participants. Data are given as mean ± (standard deviation).

Parameters	Main experiment	Control experiment	*P*-value
Age (years)	18.1 (±1.0)	17.8 (±1.3)	0.77
Height (m)	1.73 (±0.7)	1.74 (±4.8)	0.81
Body mass (kg)	63.5 (±8.5)	64.2 (±7.7)	0.53
Years of practice (years)	10.2 (±1.4)	9.9 (±1.6)	0.61
Male personal best 100 m (s)	56.88 (±1.91)	57.14 (±3.35)	0.62
Female personal best 100 m (s)	62.18 (±1.94)	62.96 (±0.36)	0.63
Male FINA points	493 (±32)	483 (±48)	0.42
Female FINA points	527 (±30)	523 (±12)	0.68

The study was conducted in accordance with the 2013 revision of the Declaration of Helsinki on human experimentation, which was approved by the local ethics committee of the University of Genoa (Comitato Etico per la Ricerca di Ateneo, Genoa, Italy, n° 2020/21). Subjects participated in this study after giving their written informed consent. In the case of underage subjects, written consent was obtained from their parents.

### 2.2 Main experiment

**Figure 1:**
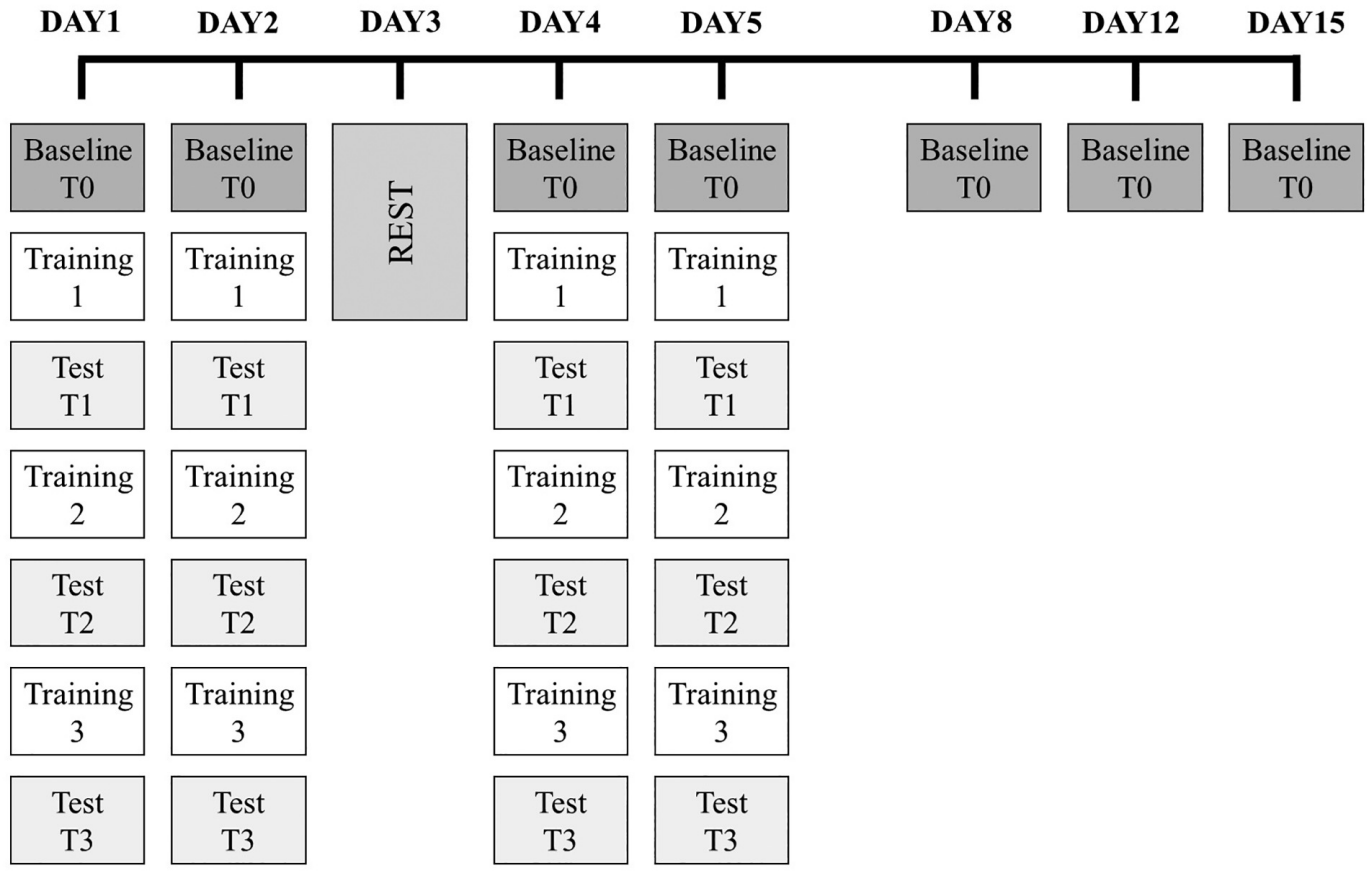
Experimental design. The entire experimental protocol lasted 15 days. Dark grey boxes are the baseline evaluations (T0), which took place at the beginning of each experimental day, during which swimmers completed a 1 × 100 m front crawl time trial at a pace corresponding to their aerobic intensity. This test was also repeated during the three test sessions (T1, T2, and T3; light grey boxes). Each test session was performed after a training session (Training 1, Training 2, and Training 3; white boxes) that consisted of three 2 × 100 m front crawl trials during which the participants were asked to swim in synchrony with the audio feedback provided by a waterproof metronome. Tests and training were performed on DAY1, DAY2, DAY4, and DAY5. On DAY8, DAY12, and DAY15, only a baseline evaluation (T0) was executed.

The experiment was performed during a specific phase of swimming training. All the sessions were conducted in a 25 m indoor swimming pool with a water temperature of 27°C. Before the test, swimmers performed a moderate intensity 1,000 m warm-up swim (35). During the first week, the athletes participated in 4 experimental days (DAY1, DAY2, DAY4, and DAY5) during which they performed both training and test sessions and had 1 rest day (DAY3). The test sessions were also repeated in the following weeks on DAY8, DAY12, and DAY15 as follow-up assessments (Figure 1). This protocol was inspired by the study of Bonassi et al. (32). that tested the fast and slow learning phases and retention of motor skills.

Before the first evaluation, the swimmers were introduced to the metronome audio feedback but were not familiarized with the task. During the baseline evaluation (T0), which took place at the beginning of each day throughout the whole experiment, the swimmers were required to complete a 1 × 100 m front crawl time trial at a pace corresponding to their aerobic intensity, i.e., the pace that a swimmer can maintain for longer distances/repetitions (13). The intensity required for each 100 m trial was defined by Touretski as 80%–90% of the swimmer's critical speed (36, 37). The critical speed of each swimmer was calculated as 80% of their best 100 m time, according to the results of Barden and Kell's study (38). In the study, the authors tested different distances to calculate the critical speed and measured the time taken to complete each trial. They concluded that the swimmer's critical speed was approximately 80% of their best 100-m time.

This calculation allowed us to determine the swimming time (target time, TT) to complete the time trials. On DAY1, DAY2, DAY4, and DAY5, after T0, participants completed training sessions. Each training session consisted of three 2 × 100 m front crawl trials (Training 1, Training 2, and Training 3) during which the participants were asked to swim in synchrony with the audio feedback provided by a waterproof metronome (Tempo Trainer Pro, Finis) positioned under the swimming cap. During turns, the metronome sound was timed to coincide with the moment their feet touched the wall, while during the final approach, the sound was synchronized with their hand touching the wall at the finish. The metronome's audio feedback was designed to help participants maintain the predefined swimming pace. The optimal time interval between metronome audio feedback (M25) was identified as the time required to swim from one side of the pool to the other (25 m) and it was calculated as the TT divided by 4 (28). Each training session was followed by a test, which included a 1 × 100 m front crawl trial without a metronome (T1, T2, and T3). The participants were blinded to their swimming times during baseline evaluations and training sessions. The experimenter timed each trial using a stopwatch (3X300M Stopwatch, Finis).

### 2.3 Control experiment

The aim of the control experiment was to assess whether verbal feedback on the time trial (100 m), usually given by the coach at the end of the training sessions, produced similar results to the use of a real-time pacing tool such as a metronome. In the control experiment, the same methodologies were followed as in the main experiment, but only on DAY1 and DAY2. The athletes performed both training and test sessions across these 2 days. Each day began with a baseline evaluation (T0), where participants completed a 1 × 100 m front crawl time trial at a pace corresponding to their aerobic intensity. However, during the training sessions on both days, instead of swimming in sync with a metronome, the coach verbally informed the swimmers of their final 100 m time at the end of each trial, simulating typical training conditions where a coach provides the time after the swim is completed. Each training session still consisted of three 2 × 100 m front crawl trials (Training 1, Training 2, and Training 3), but no audio feedback was provided during the swims. Following each training session, the participants completed a test session with a 1 × 100 m front crawl trial without any feedback (T1, T2, and T3). The participants were blinded to their swimming times during the baseline evaluations and training sessions, and the experimenter timed each trial using a stopwatch (3X300M Stopwatch, Finis).

### 2.4 Data and statistical analysis

The total time for the 100 m trial (T100) and the time interval for each lap (T25) were measured. The outcome parameters used in both experiments to evaluate the performance of the trials included (1) temporal error over 100 m (Err100), obtained as the time difference between TT and T100 swum during each trial, providing information on the athlete's ability to complete the trial in TT; (2) temporal error over 25 m (Err25), calculated as the time difference between M25 and T25, averaged over the laps, describing the athlete's ability to swim in sync with the metronome; and (3) coefficient of variation (CV) of Err25, calculated as the standard deviation of T25 divided by the mean of T25, quantifying the swimmer's performance variability over the four laps. Normality was checked by means of the Shapiro–Wilk test. In the main experiment, all the outcome parameters were normally distributed. Repeated measures ANOVAs with TIME (four levels: T0, T1, T2, and T3) as a within-subject factor were applied to the outcome parameters to investigate the sensorimotor adaptation after the first training session (namely, at the end of DAY1).

Repeated measures ANOVAs with TIME (two levels: T0 and T3) and DAY (four levels: DAY1, DAY2, DAY4, and DAY5) as within-subject factors were applied to the outcome parameters to evaluate the sensorimotor adaptation after 1 week of training. Finally, to assess retention, ANOVAs with DAY (four levels: DAY1, DAY8, DAY12, and DAY15) as a within-subject factor were applied to compare the outcome parameters at T0.

Newman–Keuls *post hoc* tests were applied in the case of a significant interaction. Partial eta square (p*η*^2^) was used to quantify effect size. Data in text are expressed as mean ± standard deviation.

In the control experiment, Err100 and Err25 were not normally distributed, while CV was normally distributed. To investigate the sensorimotor adaptation after the first training session (namely, at the end of DAY1), Friedman tests with TIME (four levels: T0, T1, T2, and T3) as a factor, followed by its *post hoc* test, were applied to Err100 and Err25. Kendall's *W* was used to quantify the effect size (39). A repeated measures ANOVA with TIME (four levels: T0, T1, T2, and T3) as a within-subject factor was applied to CV. Newman–Keuls *post hoc* tests were applied in the case of a significant TIME effect. Partial eta square (p*η*^2^) was used to quantify effect size.

To evaluate the sensorimotor adaptation after 2 days of training, Friedman tests, with TIME (two levels: T0 and T3) and DAY (two levels: DAY1 and DAY2) as factors, followed by its *post hoc* test, were applied to Err100 and Err25. Kendall's *W* was used to quantify the effect size. A repeated measures ANOVA with TIME (two levels: T0 and T3) and DAY (two levels: DAY1 and DAY2) as within-subject factors was applied to CV. Newman–Keuls *post hoc* tests were applied in case of significant interaction. Partial eta square (p*η*^2^) was used to quantify the effect size.

In the text, normally distributed data are expressed as mean ± standard deviation, while non-normally distributed data are given as median and interquartile range. The significance level was set at *p* < 0.05. Statistical analyses were performed with SPSS Statistics 26 software.

## 3 Results

### 3.1 Main experiment

**Figure 2:**
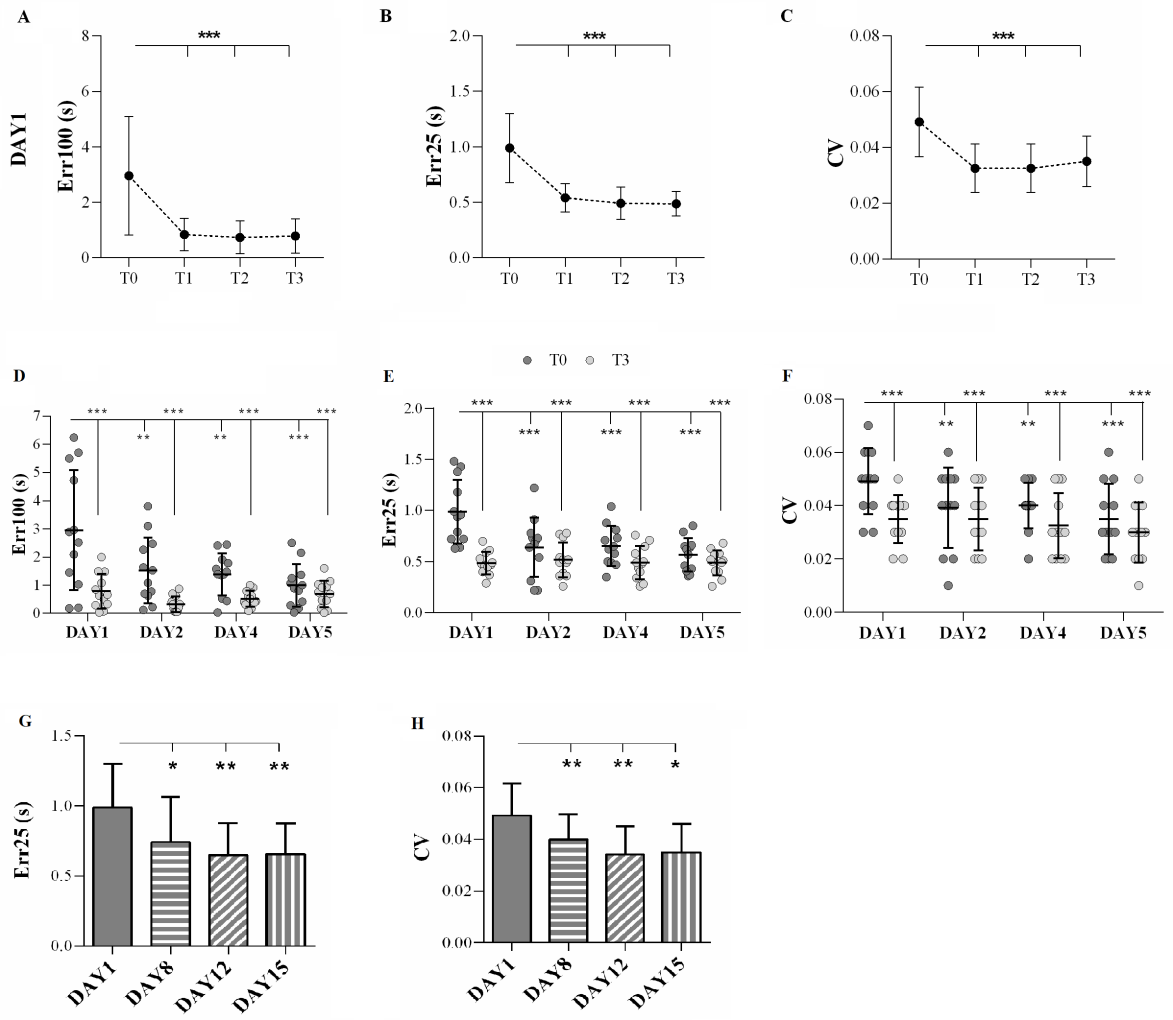
Main experiment. **(A–C)** The results of the analysis on DAY 1. Temporal error over 100 m (Err100, **A**), temporal error over 25 m (Err25, **B**), and coefficient of variation (CV, **C**) after the first day of training (DAY1) were evaluated during the test sessions (T0, T1, T2, and T3). Circles indicate the mean value, and the error bars represent the standard deviation. **(D–F)** The results of the comparisons among DAY1, DAY2, DAY4, and DAY5. Err100 **(D)**, Err25 **(E),** and CV **(F)** were measured during the baseline evaluation (T0; dark grey circles) and the last test session (T3; light grey circles) on each day over 1 week of training. The horizontal lines indicate the mean value, and the error bars represent the standard deviation. **(G)** Err25 and **(H)** CV measured during baseline evaluation (DAY1) and follow-up assessments (DAY8, DAY12, and DAY15). The columns indicate the mean value, and the error bars represent the standard deviation. *p < 0.05, **p < 0.01, ***p < 0.001.

Numerical results are reported in Table 2. After the first day of training, the repeated measures ANOVA revealed a main effect of TIME on Err100 (*F*_3,33_ = 9.46, *p* < 0.001, p*η*^2^ = 0.46), Err25 (*F*_3,33_ = 18.63, *p* < 0.001, p*η*^2^ = 0.63), and CV (*F*_3,33_ = 10.13, *p* < 0.001, p*η*^2^ = 0.49). The *post hoc* analysis showed that Err100 was higher at T0 than at T1, T2, and T3 (always *p* < 0.001). Similar patterns were observed for Err25, where the *post hoc* analysis indicated that Err25 was higher at T0 compared to T1, T2, and T3 (always *p* < 0.001). Consistently, the *post hoc* analysis of CV values showed that CV was higher at T0 than at T1, T2, and T3 (always *p* < 0.001). The results are represented in Figure 2.

**Table 2:** Results of the main experiment on the different days and test epochs (T0, T1, T2, and T3).

Parameters	DAY1				DAY2		DAY4		DAY5		DAY8	DAY12	DAY15
Main experiment													
	T0	T1	T2	T3	T0	T3	T0	T3	T0	T3	T0	T0	T0
Err100 (s)	2.96 ± 2.31	0.84 ± 0.58	0.73 ± 0.59	0.78 ± 0.62	1.53 ± 0.34	0.32 ± 0.08	1.39 ± 0.22	0.53 ± 0.08	1 ± 0.22	0.71 ± 0.15	2.01 ± 0.48	1.69 ± 0.36	1.84 ± 0.39
Err25 (s)	0.99 ± 0.31	0.54 ± 0.13	0.49 ± 0.15	0.49 ± 0.11	0.64 ± 0.08	0.52 ± 0.05	0.65 ± 0.06	0.49 ± 0.05	0.57 ± 0.05	0.49 ± 0.04	0.72 ± 0.08	0.63 ± 0.06	0.64 ± 0.05
CV	0.05 ± 0.011	0.35 ± 0.009	0.32 ± 0.008	0.34 ± 0.009	0.04 ± 0.005	0.03 ± 0.003	0.04 ± 0.002	0.03 ± 0.004	0.03 ± 0.003	0.03 ± 0.003	0.04 ± 0.002	0.03 ± 0.002	0.03 ± 0.003
Control experiment													
Err100 (s)	1.84 [0.86, 2.94]	1.36 [0.72, 1.99]	0.79 [0.41, 1.63]	0.59 [0.47, 0.86]	1.66 [1.14, 2.35]	0.44 [0.09, 0.60]	—	—	—	—	—	—	—
Err25 (s)	0.75 [0.68, 0.86]	0.84 [0.50, 1.02]	0.72 [0.59, 0.78]	0.58 [0.54, 0.83]	0.78 [0.65, 0.87]	0.56 [0.46, 0.82]	—	—	—	—	—	—	—
CV	0.05 ± 0.004	0.04 ± 0.004	0.04 ± 0.003	0.04 ± 0.004	0.04 ± 0.004	0.04 ± 0.004	—	—	—	—	—	—	—

Data are given as mean ± standard error or median [interquartile range], according to the type of statistical analysis used.

Err100, temporal error over 100 m; Err25, temporal error over 25 m; CV, coefficient of variation of Err25.

Statistical analysis of data evaluating the effects of 1 week of training on Err100 showed a main effect of DAY (*F*_3,33_ = 6.68, *p* = 0.001, p*η*^2^ = 0.38). The *post hoc* analysis indicated that Err100 was higher on DAY1 compared to the other days (always *p* < 0.01). Furthermore, a main effect of TIME (*F*_1,11_ = 31.74, *p* < 0.001, p*η*^2^ = 0.74) was found, indicating that Err100 at T0 was worse than at T3 (*p* < 0.001). A DAY × TIME interaction emerged (*F*_3,33_ = 3.85, *p* = 0.018, p*η*^2^ = 0.26). The *post hoc* analysis showed that Err100 at T0 acquired on DAY1 was higher than in all other acquisitions (always *p* < 0.01) (Figure 2D).

A main effect of DAY was also found on Err25 (*F*_3,33_ = 6.36, *p* = 0.002, p*η*^2^ = 0.37), and the *post hoc* analysis showed that Err25 was highest on DAY1 compared to the other days (always *p* < 0.01). Moreover, a main effect of TIME was found (*F*_1,11_ = 35.45, *p* < 0.001, p*η*^2^ = 0.77), indicating that Err25 was higher at T0 than at T3 (*p* < 0.001). A DAY × TIME interaction was also found (*F*_3,33_ = 9.71, *p* < 0.001, p*η*^2^ = 0.47) and the *post hoc* analysis showed that Err25 was higher at T0 on DAY1 than on all other acquisition periods (always *p* < 0.01) (Figure 2E).

Finally, statistical analysis of CV showed a main effect of DAY (*F*_3,33_ = 5.55, *p* < 0.0001, p*η*^2^ = 0.34). The *post hoc* analysis highlighted that CV was lower on DAY5 compared to DAY1 (*p* = 0.002). Moreover, a main effect of TIME (*F*_1,11_ = 30.31, *p* < 0.001, p*η*^2^ = 0.73) was found, indicating that T0 was higher than T3 (*p* < 0.001). A DAY × TIME interaction was also obtained (*F*_3,33_ = 2.97, *p* = 0.046, p*η*^2^ = 0.21), and the *post hoc* analysis revealed that CV was higher in T0 of DAY1 with respect to all the other periods (always *p* < 0.01) (Figure 2F).

Concerning the follow-up assessment, the statistical analysis showed a main effect of DAY on Err25 (*F*_3,33_ = 6.03, *p* = 0.002) and CV (*F*_3,33_ = 10.40, *p* < 0.0001). No difference was observed in Err100 (*F*_3,33_ = 1.64, *p* = 0.20). The *post hoc* analysis for Err25 indicated that its baseline value was highest on DAY1 than on any of the other days (always *p* < 0.01). The CV *post hoc* analysis showed that the baseline on DAY1 was the highest compared to the other baseline values (always *p* < 0.001). The results are displayed in Figures 2G,H.

### 3.2 Control experiment

The results of the statistical analyses on the outcome parameters after the first day of training showed no effect of TIME on Err100, Err25, and CV.

A significant effect was found by the Friedman test on Err100 [*χ*^2^(12, 3) = 12.70, *p* = 0.005, *W* = 0.6]. The *post hoc* tests showed that Err100 at T3 on DAY2 was lower compared to T0 on DAY1 (*p* = 0.04) and T0 in DAY2 (*p* = 0.005).

No significant effects were found on the Err25 and CV values.

## 4 Discussion

Given the importance of acquiring the optimal training pace for improving performance, the aim of this study was to investigate the stages of adaptation to an individually defined pace imposed by a metronome during swimming training sessions. In particular, the study focused on the assessment of the time required by the athletes to adopt the new pace when tested without metronome guidance.

The main experiment showed that (I) the participants improved their performance, as evidenced by reductions in Err100, Err25, and CV after the first day of training; (II) the athletes maintained their performance gains, with Err100, Err25, and CV on DAY1-T0 being higher compared to subsequent test sessions; and, (III) after 1 week of training, the participants maintained the new pace during the completion of the trials, as evidenced by Err25 and CV values that were lower than those at DAY1-T0.

Motor adaptation refers to the behavioral changes that involve adjusting how a previously well-practiced action is executed (29). In a similar vein, the use of a metronome in the present study acted as a perturbation to their own movement tempo, requiring a motor adaptation of the athletes' swimming technique in order to move in synchrony with the acoustic cue. Analogous to the adjustments observed in motor adaptation paradigms, where participants modify their actions in response to changes in the environment (29), the athletes exhibited a dynamic recalibration of their swimming pace. Typically, the extent of correction from one trial to the next depends on the size of the error (29). For instance, after introducing a perturbation, i.e., a metronome sound, movement errors are substantial, prompting participants to generate a relatively larger correction, as observed in our experiment during the first training day between T0 and T1. As learning progresses, the errors in both 100 m and 25 m decreased, leading to correspondingly smaller corrections, which were also observed between T2 and T3 on DAY1 (29, 40). Furthermore, CV exhibited notable reductions, indicating a reduced variability of the time needed to complete a lap, which can be interpreted as an improvement in the participants' timing consistency (24,34). These findings highlight the remarkable impact of using a metronome in swimming for just one training session, showing its effectiveness in rapidly improving training performance and promoting independence in maintaining training pace. With the subsequent training days outlined in the experimental design (DAY2, DAY4, and DAY5), learning reached saturation, resulting in a performance plateau, as described in the literature by Albert et al. (41). Finally, with regard to the retention phase, the results showed that the improvements in the participants' single lap performance (Err25) and the reduction in variability (CV) were maintained at the follow-up assessments up to DAY15, i.e., 10 days after the end of the training. These findings underline the effectiveness of metronome-guided training in inducing a sustained improvement in training performance over time. One possible neurophysiological explanation for these improvements concerns the activity of the cerebellum. Indeed, numerous lines of evidence have shown that the cerebellum plays a critical role in motor adaptation (42, 43), and, in particular, in learning from sensory prediction errors (44, 45). It is speculated that the cerebellum receives multiple feedbacks from the sound that the metronome provided each lap, allowing it to make multiple estimates of the errors. As a result of these error-based adaptation mechanisms, the cerebellum may have had the opportunity to make multiple online corrections, leading to improved temporal accuracy. This explanation is supported by the results of the control experiment, where participants were verbally informed of their final 100 m time only after the swim attempt. The results did not reveal significant improvements by the end of DAY1, unlike what was observed after the metronome-guided training. The only notable improvement observed in the control experiment was in the Err100 at T3 on DAY2. This also implies that there was no consolidation between the first and second training days, indicating a slower adaptation process, possibly due to the lower amount of feedback provided to the swimmers being insufficient for the cerebellum to efficiently perform the error-based adaptation mechanism. As a consequence, the participants needed more sessions to achieve improvements with traditional training.

A point that needs to be discussed is that, in the present study, the metronome sound was adjusted to coincide with the time required to swim from one side of the pool to the other, thus leaving swimmers free to regulate their stroke rate. This choice was motivated by the aim of this study, which was to use a simple and cost-effective tool, accessible to all coaches and athletes, to impose a fixed lap time. Naturally, this approach entails some variability within the lap, as stroke rate and stroke length were not directly controlled. However, to achieve such control using only a metronome, we would have needed two separate devices—one set to stroke rate and the other to lap time—a setup considered impractical. In fact, to achieve this, a study by Franken et al. (46) combined a metronome with a visual light strip, a solution that is both more expensive and more complex to implement in daily training. Furthermore, in the study of Puce et al., using a metronome in such a way allowed them to unveil differences among different swimming drafting configurations, proving to be a useful methodology to establish the rhythm in swimming (28). It would be interesting in future research to replicate the present paradigm with controlled stroke rates to examine its effect on pace adaptation mechanisms. In general, it should be noted that both stroke rate and stroke amplitude could provide valuable insights into a swimmer’s performance (47). These parameters were not assessed in the present study, which represents a limitation.

In the context of aerobic training, these findings are particularly important as they highlight the role of the metronome as a key tool in improving swimmers' training performance more rapidly than with traditional training methods. By adapting the swimming performance to the pace of the metronome, which was tailored to the individual swimmer’s characteristics, swimmers can fine-tune the intensity of their aerobic workouts. The metronome serves as a precise guide to help the athletes maintain a consistent intensity throughout the training session. The reduction in variability shown in the present study reflects an increased accuracy in adhering to the imposed motor timing, resulting in reduced uncertainty during aerobic training and thus potentially improving the overall performance. This practice fosters continuous learning during aerobic training. The use of the metronome can be complemented with other targeted training techniques to optimize aerobic performance. A synergistic approach that combines tools and methods contributes to a more comprehensive and personalized training regimen.

## 5 Conclusions

The present study successfully delved into the stages of adaptation to new motor timing during metronome-guided swim training and showed that, after the first day of training, swimmers successfully adapted to the imposed pace and maintained this pace up to 10 days after the end of training. This work lays the groundwork for future investigations that may include exploring the efficacy of metronome-guided pacing in high-intensity training, assessing the ability of athletes to learn different rhythmic patterns, and investigating the transferability of adaptive skills to non-guided sessions. These results advance our understanding of metronome-based training methodologies and their potential applications in optimizing athletic performance.

## Data Availability

The raw data supporting the conclusions of this article will be made available by the authors, without undue reservation.
